# Processing of social and monetary rewards in autism spectrum disorders

**DOI:** 10.1192/bjp.2022.157

**Published:** 2023-03

**Authors:** Sarah Baumeister, Carolin Moessnang, Nico Bast, Sarah Hohmann, Pascal Aggensteiner, Anna Kaiser, Julian Tillmann, David Goyard, Tony Charman, Sara Ambrosino, Simon Baron-Cohen, Christian Beckmann, Sven Bölte, Thomas Bourgeron, Annika Rausch, Daisy Crawley, Flavio Dell'Acqua, Guillaume Dumas, Sarah Durston, Christine Ecker, Dorothea L. Floris, Vincent Frouin, Hannah Hayward, Rosemary Holt, Mark H. Johnson, Emily J. H. Jones, Meng-Chuan Lai, Michael V. Lombardo, Luke Mason, Bethany Oakley, Marianne Oldehinkel, Antonio M. Persico, Antonia San José Cáceres, Thomas Wolfers, Eva Loth, Declan G. M. Murphy, Jan K. Buitelaar, Heike Tost, Andreas Meyer-Lindenberg, Tobias Banaschewski, Daniel Brandeis, the EU-AIMS LEAP Group

**Affiliations:** Department of Child and Adolescent Psychiatry and Psychotherapy, Central Institute of Mental Health, Medical Faculty Mannheim, University of Heidelberg, Mannheim, Germany; Department of Psychiatry and Psychotherapy, Central Institute of Mental Health, University of Heidelberg, Mannheim, Germany; Department of Child and Adolescent Psychiatry and Psychotherapy, Central Institute of Mental Health, Medical Faculty Mannheim, University of Heidelberg, Mannheim, Germany and Department of Child and Adolescent Psychiatry, Psychosomatics and Psychotherapy, University Hospital Frankfurt am Main, Goethe University, Frankfurt, Germany; Department of Psychology, Institute of Psychiatry, Psychology & Neuroscience, King's College London, London, United Kingdom and Department of Applied Psychology: Health, Development, Enhancement, and Intervention, University of Vienna, Vienna, Austria; Neurospin Centre CEA, Saclay, Gif sur Yvette, France; Department of Psychology, Institute of Psychiatry, Psychology & Neuroscience, King's College London, London, UK; Department of Psychiatry, University Medical Center Utrecht Brain Center, Utrecht University, Utrecht, the Netherlands; Autism Research Centre, Department of Psychiatry, University of Cambridge, UK; Donders Institute for Brain, Cognition and Behaviour, Radboud University, Nijmegen, the Netherlands and Department of Cognitive Neuroscience, Radboud University Medical Centre, Nijmegen, the Netherlands; Center of Neurodevelopmental Disorders (KIND), Centre for Psychiatry Research; Department of Women's and Children's Health, Karolinska Institutet and Child and Adolescent Psychiatry, Stockholm Health Care Services, Region Stockholm, Stockholm, Sweden and School of Allied Health, University of Western Australia, Perth, Western Australia; Institut Pasteur, Human Genetics and Cognitive Functions Unit, Paris, France; Department of Forensic and Neurodevelopmental Sciences, Institute of Psychiatry, Psychology & Neuroscience, King's College London, London, UK; Department of Forensic and Neurodevelopmental Sciences, Institute of Psychiatry, Psychology & Neuroscience, King's College London, London, UK and Sackler Institute for Translational Neurodevelopment, Institute of Psychiatry, Psychology & Neuroscience, King's College London, London, UK; Department of Child and Adolescent Psychiatry, Psychosomatics and Psychotherapy, University Hospital Frankfurt am Main, Goethe University, Frankfurt, Germany; Donders Institute for Brain, Cognition and Behaviour, Radboud University, Nijmegen, the Netherlands; Department of Cognitive Neuroscience, Radboud University Medical Centre, Nijmegen, the Netherlands and Methods of Plasticity Research, Department of Psychology, University of Zurich, Zurich, Switzerland; Department of Forensic and Neurodevelopmental Sciences, Institute of Psychiatry, Psychology & Neuroscience, King's College London, UK; Autism Research Centre, Department of Psychiatry, University of Cambridge, UK and Centre for Brain and Cognitive Development, Birkbeck, University of London, UK; Centre for Brain and Cognitive Development, Birkbeck, University of London, London, UK; Autism Research Centre, Department of Psychiatry, University of Cambridge, UK; Centre for Addiction and Mental Health and The Hospital for Sick Children, Department of Psychiatry, University of Toronto, Canada and Department of Psychiatry, National Taiwan University Hospital and College of Medicine, Taiwan; Autism Research Centre, Department of Psychiatry, University of Cambridge, UK and Laboratory for Autism and Neurodevelopmental Disorders, Center for Neuroscience and Cognitive Systems @UniTn, Istituto Italiano di Tecnologia, Italy; Centre for Brain and Cognitive Development, Birkbeck, University of London, UK; Department of Forensic and Neurodevelopmental Sciences, Institute of Psychiatry, Psychology & Neuroscience, King's College London, UK and Sackler Institute for Translational Neurodevelopment, Institute of Psychiatry, Psychology & Neuroscience, King's College London, UK; Donders Institute for Brain, Cognition and Behaviour, Radboud University, the Netherlands and Department of Cognitive Neuroscience, Radboud University Medical Centre, the Netherlands; Child and Adolescent Neuropsychiatry Program at Modena University Hospital, & Department of Biomedical, Metabolic and Neural Sciences, University of Modena and Reggio Emilia, Italy; Department of Forensic and Neurodevelopmental Sciences, Institute of Psychiatry, Psychology & Neuroscience, King's College London, UK and Instituto de Investigación Sanitaria Gregorio Marañón, Hospital General Universitario Gregorio Marañón and CIBERSAM, Spain; Donders Institute for Brain, Cognition and Behaviour, Radboud University, Nijmegen, the Netherlands; Department of Cognitive Neuroscience, Radboud University Medical Centre, Nijmegen, the Netherlands and Karakter Child and Adolescent Psychiatry University Centre, Nijmegen, the Netherlands; Department of Child and Adolescent Psychiatry and Psychotherapy, Central Institute of Mental Health, Medical Faculty Mannheim, University of Heidelberg, Mannheim, Germany; Department of Child and Adolescent Psychiatry and Psychotherapy, Psychiatric University Hospital, University of Zurich, Zurich, Switzerland and Neuroscience Center Zurich, University of Zurich and ETH Zurich, Zurich, Switzerland

**Keywords:** Autism spectrum disorder, autism traits, reward processing, fMRI, ADHD symptoms, multisite

## Abstract

**Background:**

Reward processing has been proposed to underpin the atypical social feature of autism spectrum disorder (ASD). However, previous neuroimaging studies have yielded inconsistent results regarding the specificity of atypicalities for social reward processing in ASD.

**Aims:**

Utilising a large sample, we aimed to assess reward processing in response to reward type (social, monetary) and reward phase (anticipation, delivery) in ASD.

**Method:**

Functional magnetic resonance imaging during social and monetary reward anticipation and delivery was performed in 212 individuals with ASD (7.6–30.6 years of age) and 181 typically developing participants (7.6–30.8 years of age).

**Results:**

Across social and monetary reward anticipation, whole-brain analyses showed hypoactivation of the right ventral striatum in participants with ASD compared with typically developing participants. Further, region of interest analysis across both reward types yielded ASD-related hypoactivation in both the left and right ventral striatum. Across delivery of social and monetary reward, hyperactivation of the ventral striatum in individuals with ASD did not survive correction for multiple comparisons. Dimensional analyses of autism and attention-deficit hyperactivity disorder (ADHD) scores were not significant. In categorical analyses, *post hoc* comparisons showed that ASD effects were most pronounced in participants with ASD without co-occurring ADHD.

**Conclusions:**

Our results do not support current theories linking atypical social interaction in ASD to specific alterations in social reward processing. Instead, they point towards a generalised hypoactivity of ventral striatum in ASD during anticipation of both social and monetary rewards. We suggest this indicates attenuated reward seeking in ASD independent of social content and that elevated ADHD symptoms may attenuate altered reward seeking in ASD.

## Background

Altered reward processing has been proposed to underlie the challenges that individuals with autism spectrum disorder (ASD) face in social interactions. The social motivation hypothesis postulates that individuals with ASD from early in development do not perceive social stimuli to be as rewarding as typically developing individuals, which may have an impact on the development of social learning and social skills.^1^ Newer models extending atypicalities beyond social stimuli to restricted and repetitive behaviours and interests stimuli have recently been proposed.^2,3^

Neurobiological evidence in favour of a social motivation atypicality is, however, mixed.^3–5^ To assess atypical motivation, reward processing is commonly investigated during the anticipation of a potential reward (‘wanting’), the delivery of the reward (‘liking’) or during both phases. Further, different types of rewards can be assessed, with non-social (usually monetary) rewards being most commonly investigated across psychiatric conditions, and it has been postulated that there is a specific impact on social rewards, as detailed in the social motivation hypothesis. Supporting the concept of atypical social reward processing in ASD, one study showed reduced activation in the ventral striatum,^6^ a key region for reward processing comprising the nucleus accumbens and caudate head, compared with control participants when receiving social rewards. A similar effect was observed in another study in more dorsal parts of the striatum.^7^ However, other studies did not find functional striatal differences between ASD and typically developing individuals for social rewards during delivery^8,9^ or anticipation.^7,8^ Similarly mixed results exist for non-social rewards: although some previous studies report ventral striatum hypoactivation in individuals with ASD when receiving monetary rewards,^10–12^ this has not been found^8^ or only at uncorrected thresholds^13^ elsewhere. Results for the anticipation of monetary rewards are also inconsistent with some studies suggesting ventral striatum hypoactivation in ASD individuals see for example references ^8,12^, whereas another showed no difference between participants with ASD and typically developing participants.^7^

Some of the inconsistency of previous findings is likely because of the heterogeneity of ASD itself, but also the relatively small sample sizes examined (ranging between 13 and 39 individuals per group). Two recent meta-analyses have partly addressed the latter issue by summarising the current literature.^4,5^ Comparing individuals with ASD to typically developing individuals, the authors reported striatal hypoactivation during social as well as non-social rewards in ASD. Only Clements et al^4^ investigated anticipation and delivery phases separately. They report hypoactivation of the left caudate during anticipation of social rewards, and hyperactivation during the anticipation of non-social rewards. In contrast, during reward delivery, striatal (left nucleus accumbens and caudate) hyperactivation to social rewards and right caudate hypoactivation to non-social rewards were observed in ASD. These findings suggest opposing atypicality patterns for social and monetary reward types between reward phases and do not imply typical non-social reward processing in ASD.

Across the seven studies assessing social reward processing, caudate hypoactivation was linked (albeit only at trend level) to the severity of autistic traits as measured with the Social Responsiveness Scale (SRS). This meta-analysis was an important first step to provide a more comprehensive insight into atypical reward processing in ASD. However, the number of included studies is still small (for example only three studies allowed for the differentiation between reward phases for social reward) and should be regarded as exploratory. Further, task designs were heterogeneous, which might have increased variability in brain responses and influenced task-specific effects. Finally, although some studies included in the meta-analysis administered social and non-social reward conditions in the same sample, some only assessed one type of reward, limiting direct comparability.

On top of these, another challenge is the fact that ASD and attention-deficit hyperactivity disorder (ADHD) frequently co-occur and atypical reward processing with ventral striatum hyporesponsiveness for monetary rewards is often reported in individuals with ADHD.^14,15^ However, co-occurring ADHD or symptoms have not been examined in the majority of studies exploring reward processing in ASD (for exceptions, see references ^13,16^).

## Aims

Hence, the brain functional mechanisms underpinning reward processing in ASD remain unclear. We therefore investigated reward-related brain responses in a large, well-powered sample of individuals with ASD. The Longitudinal European Autism Project (LEAP^17^) provides a deeply phenotyped data-set of children, adolescents and adults with and without ASD who performed a social and a monetary reward task. The task was chosen based on its ability to reliably elicit ventral striatum reward signalling^18^ and allows for the analysis of both reward anticipation and reward delivery phases. We comprehensively assessed differences in reward signalling based on clinical diagnosis as well as dimensional autistic traits. Based on the recent meta-analysis,^4^ compared with typically developing individuals, we hypothesised that neurofunctional responses in the ventral striatum would show a pattern of increased activity in ASD during monetary reward anticipation and reduced activity during social reward anticipation – and the opposing pattern during reward delivery. We expected to observe this pattern in categorical case–control comparisons as well as in dimensional analyses based on autism traits. Further, based on previous findings in the, to our knowledge, only study exploring reward processing in individuals with autism, ADHD, and co-occurring autism and ADHD,^16^ we hypothesised an additive effect of ADHD co-occurrence, with reward processing being most severely altered in autistic individuals with elevated ADHD symptoms.

## Method

### Experimental procedure

#### Participants

In the LEAP study, 437 individuals with ASD and 300 typically developing individuals, aged between 6 and 30 years, underwent comprehensive clinical, cognitive and magnetic resonance imaging (MRI) assessment at one of six study sites: Institute of Psychiatry, Psychology and Neuroscience, King's College London, UK (KCL); Autism Research Centre, University of Cambridge, UK (UCAM); Radboud University Nijmegen Medical Centre, the Netherlands (RUNMC); University Medical Centre Utrecht, the Netherlands (UMCU); Central Institute of Mental Health, Mannheim, Germany (CIMH); University Campus Bio-Medico of Rome, Italy (UCBM).^17^

The authors assert that all procedures contributing to this work comply with the ethical standards of the relevant national and institutional committees on human experimentation and with the Helsinki Declaration of 1975, as revised in 2008. All procedures involving patients were approved by the local ethical committees of participating centres and written informed consent was obtained from all participants or their legal guardians (for participants <18 years). For further details about the study design we refer the reader to Loth et al,^17^ and for a comprehensive clinical characterisation of the LEAP cohort we refer the reader to Charman et al.^19^ For this study, the final sample consisted of *n* = 212 participants with ASD and *n* = 181 typically developing participants (see Table 1 and Supplementary Table 1 available at https://doi.org/10.1192/bjp.2022.157). Standard operating and quality control procedures leading to the final sample are detailed in the Supplementary material.

**Table 1:** Participant characteristics^a^

	ASD group	Typically developing group	Group comparison, *χ*^2^/*t (P)*
Total *n*	212	181	
Demographics			
Sex (male/female): *n*	157/55	115/66	*χ^2^*(1) = 5.072 (0.024)
Age (years): mean (s.d.), range	17.19 (5.38), 7.56–30.60	17.69 (5.64), 7.57–30.78	*t*(391) = −0.895 (0.372)
IQ (full-scale IQ): mean (s.d.), range	105.72 (4.90), 75.00–148.00	107.37 (12.50), 75.56–141.00	*t*(389.86) = −1.190 (0.235)
Handedness (right/left/ambidextrous/unknown): *n*	149/26/8/29	122/15/4/40	*χ^2^*(3) = 6.322 (0.097)
Medication use (no/yes/unknown): *n*	64/82/66	72/12/97	*χ^2^*(2) = 56.400 (<0.001)
Site: CIMH/UCAM/RUNMC/KCL/UMCU/UCBM: *n*	22/29/63/55/32/11	23/24/52/28/38/16	*χ^2^*(5) = 9.383 (0.095)
fMRI quality control, mean (s.d.), range			
SID Mean FD (in mm)	0.13 (0.07), 0.03–0.41	0.11 (0.06), 0.03–0.34	*t*(391) = 2.458 (0.014)
SID Volumes with FD >0.5 mm (in %)	2.35 (3.96), 0–18.24	1.67(3.34), 0–16.22	*t*(390.96) = 1.858 (0.064)
SID Signal-to-noise ratio	9.76 (1.25), 6.28–12.51	9.90 (1.23), 6.49–13.10	*t*(391) = −1.057 (0.291)
MID Mean FD (in mm)	0.14 (0.07), 0.03–0.36	0.12 (0.07), 0.03–0.41	*t*(390.21) = 1.910 (0.057)
MID Volumes with FD >0.5 mm (in %)	2.83 (4.37), 0–19.59	2.06 (3.57), 0–16.89	*t*(389.00) = 1.939 (0.053)
MID Signal-to-noise ratio	9.83 (1.38), 6.08–13.62	10.00 (1.41), 6.40–14.08	*t*(391) = −1.225 (0.221)
*Clinical characteristics*			
ADI-R, mean (s.d.), range			
Reciprocal social interaction	15.45 (6.59), 0–29.00		
Communication	12.47 (5.69), 0–26.00		
RRB	3.93 (2.67), 0–12.00		
ADOS-2 (CSS), mean (s.d.), range			
Social affect	5.71 (2.52), 1.00–10.00		
RRB	4.28 (2.46), 1.00–10.00		
Total	4.85 (2.60), 1.00–10.00		
SRS-2, mean (s.d.), range			
Raw score	84.69 (30.45), 20.00–163.00	24.62 (15.03), 1.00–87.00	*t*(291.04) = 23.784 (<0.001)
*T*-score	68.62 (12.20), 43.00–90.00	45.77 (5.37), 37.00–66.00	*t*(274.80) = 23.150 (<0.001)
ADHD research diagnosis^b^ (ADHD/noADHD/missing): *n*	69/118/25	11/130/40	*χ^2^*(1) = 36.905 (*<*0.001)
DAWBA comorbidities, mean (s.d.), range			
ADHD symptoms	1.80 (1.57), 0–5.00	0.28 (0.82), 0–4.00	*t*(214.96) = 9.386 (<0.001)
Anxiety symptoms	2.60 (1.31), 0–5.00	0.96 (0.69), 0–4.00	*t*(318.90) = 11.888 (<0.001)
Depression symptoms	0.84 (1.20), 0–5.00	0.24 (0.52), 0–2.00	*t*(275.46) = 5.632 (<0.001)

ADHD, attention-deficit hyperactivity disorder; ASD, autism spectrum disorder; CIMH, Central Institute of Mental Health, Mannheim, Germany; FD, framewise displacement; fMRI, functional magnetic resonance imaging; KCL, Institute of Psychiatry, Psychology and Neuroscience, King's College London, UK; MID monetary incentive delay task; RUNMC, Radboud University Nijmegen Medical Centre, the Netherlands; SID social incentive delay task; UCAM, Autism Research Centre, University of Cambridge, UK; UCBM, University Campus Bio-Medico of Rome, Italy; UMCU, University Medical Centre Utrecht, the Netherlands.

a. ADI-R: Autism Diagnostic Interview-Revised. Scores were computed for reciprocal interaction (social interaction), communication, and restrictive, repetitive stereotyped behaviours and interests (RRB). ADOS-2 (CSS): Autism Diagnostic Observation Schedule 2. Calibrated severity scores were computed for social affect, restricted and repetitive behaviours (RRB) and the overall total score. SRS-2: Social Responsiveness Scale-2. Total raw and total *T*-scores (gender + age normalised) are reported. The raw SRS-2 scores were used in our analyses. Self-rated scores were used when parent-rated scores were not available. Symptoms of ADHD, depression and anxiety were assessed with the Development and Well Being Assessment (DAWBA), generating six levels (ordinal scores 0 to 5) of prediction of the probability of a disorder (~0.1%, ~0.5%, ~3%, ~15%, ~50%, >70%).

b. ADHD research diagnosis was based on applying DSM-V criteria to symptom scores in the parent- and self-rated ADHD rating scale.

#### Clinical measures

Participants in the ASD group had an existing clinical diagnosis of ASD according to the DSM-IV/ICD-10 or DSM-5 criteria. ASD symptoms were comprehensively assessed using the Autism Diagnostic Interview-Revised (ADI-R^20^) and Autism Diagnostic Observation Schedule 2nd edition (ADOS-2^21^) within the ASD group. We used the total raw score on the Social Responsiveness Scale 2nd edition (SRS-2^22^) to assess continuous autism traits, which was available across the study sample. Parent-rated scores were collected for ASD and typically developing individuals, except for typically developing adults where only the self-report was assessed. We used self-rated scores wherever parent-rated scores were not available. Parent- or self-report of a psychiatric disorder was an exclusion criterion for the typically developing group. Information on the presence of a confirmed diagnosis of ADHD was not available in our sample. As a proxy, we estimated diagnostic status by applying DSM-5 criteria based on symptom scores collected with the parent- and self-rated ADHD DSM-5 rating scales.^23^

#### Experimental paradigm

**Fig. 1:**
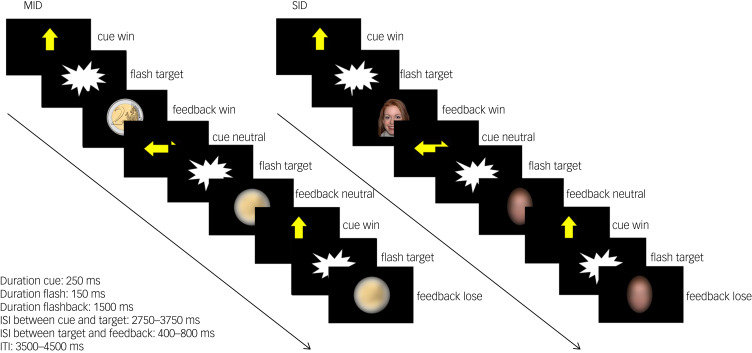
Task design of the monetary incentive delay task (MID) and social incentive delay task (SID). Participants were asked to give a speeded response (button press) to a visual target (screenflash). A cue arrow pointing upwards indicated the possibility to obtain a reward if responses were given within a predefined response time window (win trial). No reward option was given in trials preceded by a horizontal cue arrow (neutral trial). Sufficiently fast responses on win trials were followed by the presentation of a 2€/£2 coin in the MID task and a smiling female face in the SID task as feedback. Blurred control stimuli were presented in neutral trials and as feedback following slow responses in win trials. Cue presentation represents reward anticipation phase, and feedback presentation represents reward delivery phase. Note that the feedback presentation was temporally decoupled from the target presentation but not from the button press. A black screen was presented during interstimulus intervals (ISI) and intertrial intervals (ITI). In total, 15 win trials and 15 neutral trials were presented in a pseudorandomised order during each task. Total task duration was 5 min per task.

We adapted a social incentive delay (SID) and a monetary incentive delay (MID) task^7^ as part of a reliable task battery.^18,24,25^ For details see Fig. 1 and Supplementary material. SID and MID were collected as separate paradigms and combined during data analysis. SID was always presented first, followed by MID. The functional magnetic resonance imaging (fMRI) scanning session was preceded by a training session outside the MRI to ensure that all participants understood the task.

#### fMRI data acquisition

fMRI data were acquired on 3T scanners from different manufacturers (General Electric, Philips, Siemens) and harmonised as much as possible across sites (for details see Supplementary material). Functional images were acquired using a blood-oxygen-level-dependent (BOLD)-sensitive T_2_*-weighted echo-planar imaging sequence (repetition time (TR) = 2 s, echo time (TE) = 30 ms, flip angle = 80°, matrix: 64 × 64, FOV: 192 × 192 mm, in-plane resolution: 3 × 3 mm, slice thickness: 4 mm, gap: 1 mm, 28 axial slices). A total of 151 volumes were obtained for each task, oriented approximately 20° steeper than the AC-PC plane.

### Data analysis

#### fMRI data preprocessing

Image preprocessing followed standard processing routines in SPM12 (http://www.fil.ion.ucl.ac.uk/spm/), including a two-pass realignment procedure, slice time correction, registration of the functional mean image to the Montreal Neurological Institute template and spatial normalisation into standard stereotactic space, application of resulting normalisation parameters to the functional time series, resampling to 3 mm isotropic voxels and smoothing with an 8 mm full-width at half-maximum Gaussian Kernel.

#### Whole-brain level fMRI data analysis

SID and MID tasks were combined as two sessions in a general linear model (GLM) at the single-subject level (see Supplementary material for details). Within-subject effects were addressed at the subject-level by quantifying within-subject effects of condition as differential response to win cues as compared with neutral cues for reward anticipation and differential response to successful win compared with neutral feedback for reward delivery. Note that *n* = 7 individuals with ASD and *n* = 7 typically developing participants were excluded from analysis of reward delivery as they withheld responses to neutral trials. Additionally, to quantify differential reward-specific responses between tasks, a contrast image for the interaction between condition (win, neutral) and task (SID, MID) was calculated.

Based on within-subject contrasts we assessed reward-specific brain activation (within-subject effect of condition) and differential reward-specific responses between tasks (within-subject interaction condition × task) across the entire sample and tested for between-group differences. Contrast images were subjected to second-level GLMs with between-subject factor group (ASD versus typically developing) and covariates age, gender assigned at birth and site.

The impact of ADHD co-occurrence was explored in a separate model, where the ASD group was split into subgroups with (*n* = 69) and without (*n* = 118) co-occurring ADHD based on estimated diagnostic status (ASD_+ADHD_ and ASD_–ADHD_, respectively) and compared with typically developing participants while controlling for age, gender assigned at birth and site (for group characteristics, see Table 2). Typically developing individuals with elevated ADHD scores based on DSM-5 criteria were excluded from this analysis. To assess the dimensional effect of autism and ADHD traits, SRS-2 raw scores and ADHD rating scale scores were added as an additional covariate of interest in separate models. Note that the diagnostic group was accounted for in this model, ensuring that effects were not driven by differences in group means. To explore group differences on a whole-brain level, significance was defined as *P*_FWE_ < 0.05, peak-level corrected across the whole brain with a cluster threshold of *k* ≥ 5. Family-wise error correction (FEW) implemented in SPM12 is based on random field theory.

**Table 2:** Subgroup characteristics^a^

	ASD_+ADHD_ subgroup	ASD_–ADHD_ subgroup	Typically developing group	Group comparison, *χ*^2^/*F/t (P)*
Total *n*	69	118	130	
Demographics				
Sex (male/female): *n*	51/18	85/33	79/51	*χ^2^*(2) = 5.952 (0.078)
Age (years): mean (s.d.), range	15.71 (4.98), 7.98–29.25	18.30 (5.59), 7.58–30.60	17.59 (5.62), 7.57–30.78	*F*(2,314) = 4.905 (0.008)
IQ (full-scale IQ): mean (s.d.), range	103.69 (14.30), 75.00–139.00	107.33 (15.32), 75.56–148.00	108.11 (11.97), 75.56–141.00	*F*(2,314) = 2.416 (0.091)
Handedness (right/left/ambidextrous/unknown): *n*	49/14/5/1	98/11/3/6	109/15/4/2	*χ^2^*(6) = 11.573 (0.072)
Medication use (no/yes/unknown): *n*	18/31/20	41/38/39	55/4/71	*χ^2^*(4) = 54.653 (<0.001)
Site: CIMH/UCAM/RUNMC/KCL/UMCU/UCBM: *n*	6/7/23/24/6/3	10/18/38/26/19/7	15/15/42/21/26/11	*χ^2^*(10) = 13.686 (0.188)
fMRI quality control, mean (s.d.), range				
SID Mean FD (in mm)	0.14 (0.07), 0.03–0.35	0.12 (0.08), 0.03–0.41	0.11 (0.06), 0.03–0.34	*F*(2,314) = 4.171 (0.016)
SID Volumes with FD >0.5 mm (in %)	2.80 (4.03), 0–18.24	2.07 (3.99), 0–18.24	1.63 (3.32), 0–16.22	*F*(2,314) = 2.170 (0.116)
SID Signal-to-noise ratio	9.94 (1.32), 6.28–12.21	9.67 (1.17), 6.86–12.51	9.96 (1.19), 6.49–13.10	*F*(2,314) = 2.044 (0.131)
MID Mean FD (in mm)	0.15 (0.07), 0.03–0.29	0.12 (0.07), 0.03–0.36	0.12 (0.06), 0.04–0.35	*F*(2,314) = 4.100 (0.017)
MID Volumes with FD >0.5 mm (in %)	3.30 (4.40), 0–16.89	2.43 (4.14), 0–19.59	1.85 (3.30), 0–16.22	*F*(2,314) = 3.192 (0.042)
MID Signal-to-noise ratio	9.96 (1.37), 6.08–13.43	9.73 (1.31), 6.75–13.19	10.03 (1.35), 6.40–13.46	*F*(2,314) = 1.665 (0.191)
*Clinical characteristics*				
ADI-R, mean (s.d.), range				
Reciprocal social interaction	16.42 (6.51), 2.00–29.00	14.76 (6.43), 0–27.00		*t*(182) = −1.691 (0.093)
Communication	13.15 (5.37), 0–24.00	12.19 (5.91), 0–26.00		*t*(182) = −1.069 (0.274)
RRB	4.778 (2.74), 0–12.00	3.41 (2.49), 0–12.00		*t*(182) = −3.484 (0.001)
ADOS-2 (CSS), mean (s.d.), range				
Social affect	5.71 (2.78), 1.00–10.00	5.59 (2.42), 1.00–10.00		*t*(154) = −0.269 (0.788)
RRB	4.67 (2.70), 1.00–10.00	4.07 (2.37), 1.00–9.00		*t*(154) = −1.446 (0.150)
Total	5.06 (2.83), 1.00–10.00	4.65 (2.48), 1.00–10.00		*t*(154) = −0.918 (0.360)
SRS-2, mean (s.d.), range				
Raw score	104.81 (24.91), 56.00–163.00	73.48 (27.36), 20.00–151.00	22.15 (13.39), 1.00–74.00	*F*(2,307) = 340.758 (<0.001)
*T*-score	76.91 (9.94), 56.00–90.00	64.09 (10.88),43.00–90.00	44.96 (4.81), 37.00–66.00	*F*(2,307) = 324.492 (<0.001)
DAWBA comorbidities, mean (s.d.), range				
ADHD symptoms	2.92 (1.22), 0–5.00	1.00 (1.25), 0–4.00	0.14 (0.51), 0–3.00	*F*(2,187) = 96.840 (<0.001)
Anxiety symptoms	3.00 (1.33), 0–5.00	2.33 (1.24), 0–5.00	0.88 (0.62), 0–3.00	*F*(2,187) = 52.972 (<0.001)
Depression symptoms	0.92 (1.30), 0–5.00	0.78 (1.17), 0–4.00	0.19 (0.44), 0–2.00	*F*(2,187) = 7.947 (<0.001)

ADHD, attention-deficit hyperactivity disorder; ASD, autism spectrum disorder; CIMH, Central Institute of Mental Health, Mannheim, Germany; FD, framewise displacement; fMRI, functional magnetic resonance imaging; KCL, Institute of Psychiatry, Psychology and Neuroscience, King's College London, UK; MID monetary incentive delay task; RUNMC, Radboud University Nijmegen Medical Centre, the Netherlands; SID social incentive delay task; UCAM, Autism Research Centre, University of Cambridge, UK; UCBM, University Campus Bio-Medico of Rome, Italy; UMCU, University Medical Centre Utrecht, the Netherlands.

a. ADI-R: Autism Diagnostic Interview-Revised. Scores were computed for reciprocal interaction (social interaction), communication, and restrictive, repetitive stereotyped behaviours and interests (RRB). ADOS-2 (CSS): Autism Diagnostic Observation Schedule 2. Calibrated severity scores were computed for social affect, restricted and repetitive behaviours (RRB) and the overall total score. SRS-2: Social Responsiveness Scale-2. Total raw and total *T*-scores (gender + age normalised) are reported. The raw SRS-2 scores were used in our analyses. Self-rated scores were used when parent-rated scores were not available. Symptoms of ADHD, depression and anxiety were assessed with the Development and Well Being Assessment (DAWBA), generating six levels (ordinal scores 0 to 5) of prediction of the probability of a disorder (~0.1%, ~0.5%, ~3%, ~15%, ~50%, >70%).

#### Region of interest (ROI) analysis

To increase sensitivity for putative case–control differences in the ventral striatum, we performed ROI analysis within a well-established *a priori* defined bilateral mask of the ventral striatum comprising the caudate head and nucleus accumbens.^18^ Mean contrast estimates (contrasts cue win > cue neutral and successful win > neutral) for each participant and both tasks were extracted and analysed using SPSS Software package for Windows(Version 25, IBM Corp. Armonk, NY, USA). Separate repeated measures ANOVAs with the within-subject factor task (MID, SID) and between-subject factor diagnosis (typically developing, ASD) and covariates age (mean centred), gender and study sites (dummy coded), were used to assess group differences for both reward processing phases (anticipation, delivery) in the left and right ventral striatum. To correct for investigating left and right ventral striatum activity separately, the critical alpha threshold was adjusted to *P* < 0.025 based on the Bonferroni procedure. Additionally, Bonferroni-correction was applied to all *post hoc* pairwise comparisons. To assess the effect of autism and ADHD traits, SRS-2 raw scores and ADHD rating scale scores were added as an additional covariate of interest in separate models. Interaction terms between diagnosis and SRS-2 or ADHD rating scale were added as well. The impact of ADHD co-occurrence was explored in another separate model, where the between-subject factor diagnosis comprised three levels (typically developing, ASD_+ADHD_ and ASD_–ADHD_).

## Results

### Functional activation analysis

#### Reward anticipation

##### Whole-brain level analysis

**Fig. 2:**
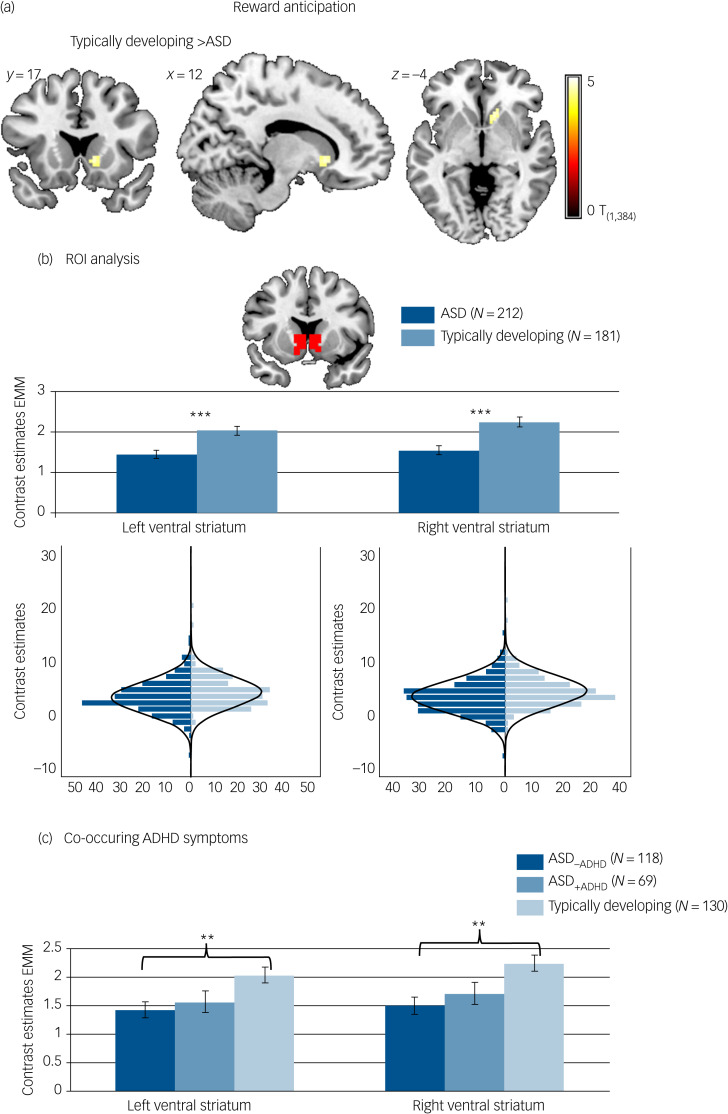
Brain activation to win compared with neutral cues. (a) Whole-brain family-wise error (FWE) corrected effect of diagnosis in the right ventral striatum. (b) Effect of diagnosis in the region of interest (ROI) analysis of the left and right ventral striatum with corresponding distribution plots. (c) ROI analysis in individuals with autism spectrum disorder (ASD) and elevated attention-deficit hyperactivity disorder (ADHD) symptoms (ASD_+ADHD_), individuals with ASD without elevated ADHD symptoms (ASD_–ADHD)_ and typically developing individuals without elevated ADHD symptoms. Location and size of ROI mask shown in red. ***P < 0.001, **P < 0.01. Error bars reflect standard error. Whole-brain results thresholded at *P*_FWE_ < 05. Distributions of ROI activation in case and control participants were compared using the Kolmogorov–Smirnov test, which suggested unequal distributions (left ventral striatum: *D*_(212,181)_ = 0.156, *P* = 0.017; right ventral striatum: *D*_(212,181)_ = 0.193, *P* = 0.001). EMM, estimated marginal means.

Reward-specific brain activation differed between diagnostic groups at the whole-brain level in the right ventral striatum (*F*_(1,384)_ = 22.84, *P*_FWE_ = 0.017, *k* = 8) during reward anticipation. A *post hoc T*-test showed that activation was reduced in participants with ASD compared with typically developing individuals. See Fig. 2(a) and Table 3 for details.

**Table 3:** Whole-brain and region of interest (ROI) effects of diagnosis on brain activation during reward anticipation and delivery^a^

	Effect of diagnosis	Direction	*k*	*x*	*y*	*z*	*F*	*P*_(FWE-corr)_
Anticipation								
Whole brain	Right caudate	Typically developing > ASD	8	12	17	−4	22.844	0.0169
ROI	Left ventral striatum: *F*_(1,384)_ = 14.163, *P* *<* 0.001, partial *η^2^* = 0.036 Right ventral striatum: *F*_(1,384)_ = 18.693, *P* *<* 0.001 partial *η^2^* = 0.046							
Delivery								
Whole brain	all *F* < 20.88, *P_FWE_* > 0.05							
ROI	Left ventral striatum: *F*_(1,370)_ = 4.829, *P* = 0.029, partial *η^2^* = 0.013 Right ventral striatum: *F*_(1,370)_ = 4.719, *P* = 0.030, partial *η^2^* = 0.013							

ASD, autism spectrum disorder.

a. Table provides test statistic for whole brain and ROI analysis for diagnosis. Voxel-level statistics were family-wise error (FWE) corrected for the number of voxels across the whole brain for each test. Significance was defined as *P*_FWE_ < 0.05 with a cluster threshold of *k* ≥ 5. For ROI analysis in the left and right ventral striatum, the critical alpha level was adjusted to *P* < 0.025 to control for multiple comparisons. Small effect sizes correspond to partial *η^2^* > 0.00995, medium effect sizes correspond to partial *η^2^* > 0.0588 and large effect sizes correspond to partial *η^2^* > 0.1379.

The effect of diagnosis was not significant for differential reward-specific responses between tasks. However, we report differences between ASD and typically developing groups in the SID and MID separately in Supplementary Figure 2 and Supplementary Tables 3 and 4 to allow for a comparison with previous studies. Task effects along with behavioural data are also reported in the Supplementary material.

##### ROI analysis

Individuals with ASD differed from typically developing individuals on average regarding reward-specific brain activation within the left (*F*_(1,384)_ = 14.163, *P* < 0.001, partial *η^2^* = 0.036) and right (*F*_(1,384)_ = 18.693, *P* < 0.001, partial *η^2^* = 0.046) ventral striatum ROI (Table 3) with reduced activation in the ASD group (left: mean 1.45, s.d. = 1.53; right: mean 1.54, s.d. = 1.58) compared with the typically developing group (left: mean 2.03, s.d. = 1.53, *d* = −0.39; right: mean 2.25, s.d. = 1.59, *d* = −0.44). See Fig. 2(b). There was no significant interaction between diagnosis and task (left ventral striatum: *F*_(1,384)_ = 2.754, *P* = 0.098, partial *η^2^* = 0.007; right ventral striatum: *F*_(1,384)_ = 2.999, *P* = 0.084, partial *η^2^* = 0.008).

#### Reward delivery

##### Whole-brain level analysis

**Fig. 3:**
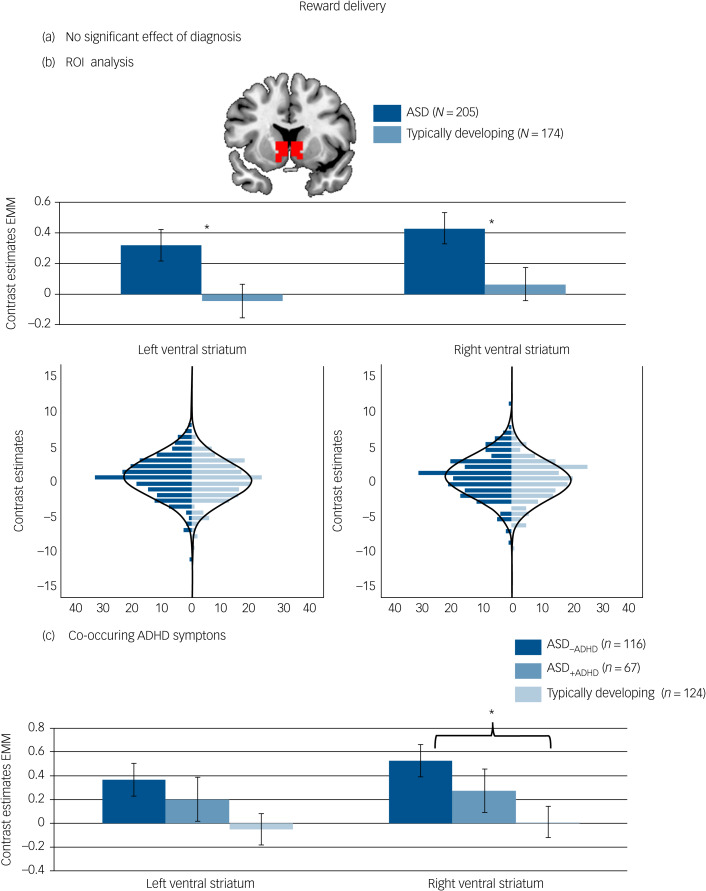
Brain activation to reward delivery. (a) No significant whole-brain family-wise error (FWE) corrected effect of diagnosis in the right ventral striatum. (b) Effect of diagnosis in the region of interest (ROI) analysis of the left and right ventral striatum with corresponding distribution plots. (c) ROI analysis in individuals with autism spectrum disorder (ASD) and elevated attention-deficit hyperactivity disorder (ADHD) symptoms (ASD_+ADHD_), individuals with ASD without elevated ADHD symptoms (ASD_–ADHD)_ and typically developing individuals without elevated ADHD symptoms. Location and size of ROI mask shown in red. *P < 0.05. Error bars reflect standard error. Whole-brain results thresholded at *P*_FWE_ < 05. Distributions of ROI activation in case and control participants were compared using the Kolmogorov–Smirnov test, which suggested no evidence for unequal distributions (left ventral striatum: *D*_(205,174)_ = 0.120, *P* = 0.134; right ventral striatum: *D*_(205,174)_ = 0.112, *P* = 0.190). EMM, estimated marginal means.

There was no significant effect of diagnostic group on reward-specific brain activation at the whole-brain level. See Fig. 3(a) and Table 3 for details.

The effect of diagnosis was not significant for differential reward-specific responses between tasks. However, we report differences between the ASD and typically developing group in the SID and MID separately in Supplementary Figure 3 and Supplementary Tables 3 and 4 to allow comparison with previous studies. Task effects along with behavioural data are also reported in the Supplementary material.

##### ROI analysis

The difference regarding reward-specific brain activation between ASD and typically developing individuals within the left (*F*_(1,370)_ = 4.829, *P* = 0.029, partial *η^2^* = 0.013) and right ventral striatum (*F*_(1,370)_ = 4.719, *P* = 0.030, partial *η^2^* = 0.013) yielded increased activation in the ASD group (Table 3) (left: mean 0.31, s.d. = 1.46; right: mean 0.42, s.d. = 1.47) compared with the typically developing group (left: mean −0.02, s.d. = 1.46, *d* = 0.23; right: mean 0.09, s.d. = 1.47, *d* = 0.23) but this did not survive correction for multiple comparisons (See Fig. 3(b)). There was no significant interaction between diagnosis and task (left ventral striatum: *F*_(1,370)_ = 1.057, *P* = 0.304, partial *η^2^* = 0.003; right ventral striatum: *F*_(1,370)_ = 1.684, *P* = 0.195, partial *η^2^* = 0.005).

#### Effect of ADHD co-occurrence

During reward anticipation, ROI analysis comparing the typically developing group, and the ASD_–ADHD_ and ASD_+ADHD_ subgroups yielded a significant effect of group in the left (*F*_(1,307)_ = 5.172, *P* = 0.006, partial *η^2^* = 0.032) and right (*F*_(1307)_ = 6.761, *P* *=* 0.001, partial *η^2^* = 0.042) ventral striatum (see Fig. 2(c)). *Post hoc* pairwise comparisons based on estimated marginal means revealed that this effect was driven by significantly reduced ventral striatum activity in the ASD_–ADHD_ subgroup compared with the typically developing group (left: *P* = 0.006, *d* = 0.40; right: *P* = 0.001, *d* = 0.46), whereas there was no significant difference between the typically developing group and the ASD_+ADHD_ subgroup (left: *P* = 0.144, *d* = 0.30; right: *P* = 0.099, *d* = 0.32) or between the two ASD subgroups (left: *P* = 1.000, *d* = −0.09; right: *P* = 1.000, *d* = −0.13).

For reward delivery, a borderline significant effect of group emerged in the right ventral striatum (*F*_(1,297)_ = 3.715, *P* *=* 0.026, partial *η^2^* = 0.024, see Fig. 3(c)) with significantly increased ventral striatum activity in the ASD_–ADHD_ subgroup compared with the typically developing group (*P* = 0.020, *d* = 0.35) and no difference between the typically developing group and the ASD_+ADHD_ subgroup (*P* = 0.741, *d* = −0.18) or between the two ASD subgroups (*P* = 0.810, *d* = 0.17). Across both reward processing stages, there was no significant effect of group on the whole-brain level and no significant interaction with the type of reward (social, monetary).

#### Dimensional effects

For both reward anticipation and delivery there was no significant main effect of autism or ADHD trait scores and no interaction between diagnosis and autism or ADHD trait scores in the ventral striatum or at the whole-brain level, see Tables 4 and 5. Autism or ADHD trait scores also showed no significant effect when analysing the typically developing and ASD groups separately.

**Table 4:** Whole-brain and region of interest (ROI) effects of autism traits on brain activation during reward anticipation and delivery^a^

	Effect of SRS-2	Interaction SRS-2 × diagnosis
Anticipation		
Whole brain	All *F* < 20.55, *P_FWE_* > 0.05	All *F* < 20.55, *P_FWE_* > 0.05
ROI	Left ventral striatum: *F*_(1,328)_ = 0.160, *P* = 0.690, partial *η^2^* = 0.000 Right ventral striatum: *F*_(1,328)_ = 0.039, *P* = 0.844, partial *η^2^* = 0.000	Left ventral striatum: *F*_(1,328)_ = 0.129, *P* = 0.722, partial *η^2^* = 0.000 Right ventral striatum: *F*_(1,328)_ = 0.570, *P* = 0.451, partial *η^2^* = 0.002
Delivery		
Whole brain	All *F* < 21.11, *P_FWE_* > 0.05	All *F* < 21.11, *P_FWE_* > 0.05
ROI	Left ventral striatum: *F*_(1,316)_ = 0.553, *P* = 0.458, partial *η^2^* = 0.002 Right ventral striatum: *F*_(1,316)_ = 0.043, *P* = 0.836, partial *η^2^* = 0.000	Left ventral striatum: *F*_(1,316)_ = 0.020 (0.887, partial *η^2^* = 0.000 Right ventral striatum: *F*_(1,316)_ = 0.229, *P* = 0.633, partial *η^2^* = 0.001

SRS-2 Social Responsiveness Scale Second Edition.

a. Table provides test statistic for whole brain and ROI analysis for autism traits. Voxel-level statistics were family-wise error (FWE) corrected for the number of voxels across the whole brain for each test. Significance was defined as *P*_FWE_ < 0.05 with a cluster threshold of *k* ≥ 5. For ROI analysis in the left and right ventral striatum, the critical alpha level was adjusted to *P* < 0.025 to control for multiple comparisons. Small effect sizes correspond to partial *η^2^* > 0.00995, medium effect sizes correspond to partial *η^2^* > 0.0588 and large effect sizes correspond to partial *η^2^* > 0.1379.

**Table 5:** Whole-brain and region of interest (ROI) effects of attention-deficit hyperactivity disorder (ADHD) traits on brain activation during reward anticipation and delivery^a^

	Effect of ADHD rating scale	Interaction ADHD rating scale × diagnosis
Anticipation		
Whole brain	All *F* < 20.89, *P_FWE_* > 0.05	All *F* < 20.90, *P_FWE_* > 0.05
ROI	Left ventral striatum: *F*_(1,317)_ = 0.668, *P* = 0.414, partial *η^2^* = 0.002 Right ventral striatum: *F*_(1,317)_ = 1.136, *P* = 0.287, partial *η^2^* = 0.004	Left ventral striatum: *F*_(1,328)_ = 0.129, *P* = 0.722, partial *η^2^* = 0.000 Right ventral striatum: *F*_(1,328)_ = 0.570, *P* = 0.451, partial *η^2^* = 0.002
Delivery		
Whole brain	All *F* < 21.39, *P_FWE_* > 0.05	All *F* < 21.42, *P_FWE_* > 0.05
ROI	Left ventral striatum: *F*_(1,307)_ = 0.143, *P* = 0.705, partial *η^2^* = 0.000 Right ventral striatum: *F*_(1,307)_ = 0.062, *P* = 0.804, partial *η^2^* = 0.000	Left ventral striatum: *F*_(1,307)_ = .068, *P* = 0.795, partial *η^2^* = 0.000 Right ventral striatum: *F*_(1,307)_ = 0.664, *P* = 0.416, partial *η^2^* = 0.002

a. Table provides test statistic for whole brain and ROI analysis for ADHD traits. Voxel-level statistics were family-wise error (FWE) corrected for the number of voxels across the whole brain for each test. Significance was defined as *P*_FWE_ < 0.05 with a cluster threshold of *k* ≥ 5. For ROI analysis in the left and right ventral striatum, the critical alpha level was adjusted to *P* < 0.025 to control for multiple comparisons. Small effect sizes correspond to partial *η^2^* > 0.00995, medium effect sizes correspond to partial *η^2^* > 0.0588 and large effect sizes correspond to partial *η^2^* > 0.1379.

#### Control analyses

Supplemental control analyses showed that results were not systematically explained by head motion, acquisition site, handedness, sex, IQ or medication status. Effects of age (linear and quadratic) were observed during reward delivery in the right superior medial frontal gyrus and the left amygdala, pallidum and (at trend-level) the ventral striatum, respectively. These effects did not differ between the ASD and typically developing groups. For reward delivery, we were not able to replicate the effect of diagnosis when investigating only female participants, only right-handed participants or when excluding participants from RUNMC or KCL. Although this likely reflects decreased statistical power because of reduced subsample sizes, it also warrants further exploration of potential sources of heterogeneity in future studies. Details on the control analyses are provided in the Supplementary material.

#### Power sensitivity analyses

*Post hoc* power sensitivity was determined using G*Power.^26^ A repeated measures ANOVA with the within-subject factor task (MID, SID) and between-subject factor diagnosis (typically developing, ASD) and *n* = 393 participants at a critical alpha of 0.025 would be sensitive to effects of partial *η^2^* = 0.0109 with 80% power. This means the study would not be able to reliably detect effects smaller than partial *η^2^* = 0.0109. The slightly reduced sample size for reward delivery with *n* = 379 participants yields 80% power at partial *η^2^* = 0.0113 and would thus not be able to reliably detect effects smaller than this.

A repeated measures ANOVA with the within-subject factor task (MID, SID) and between-subject factor diagnosis (typically developing, ASD_+ADHD_ and ASD_–ADHD_) and *n* = 317 participants at a critical alpha of 0.025 would be sensitive to effects of partial *η^2^* = .012 with 80% power. This means, the study would not be able to reliably detect effects smaller than partial *η^2^* = 0.012. The slightly reduced sample size for reward delivery with *n* = 307 participants yields 80% power at partial *η^2^* = 0.0124 and would thus not be able to reliably detect effects smaller than this. These partial *η^2^* = values all represent small effects sizes.

## Discussion

### Main findings regarding autism

In the present study, we assessed functional brain activation during monetary and social reward anticipation and delivery in a well-powered sample comprising individuals with ASD and typically developing individuals. This allowed us to examine effects of reward type during both reward processing phases. We found a reduction of ventral striatum activity during reward anticipation in individuals with ASD that did not differ between social and monetary rewards. In contrast, during reward delivery, we found that increased ventral striatum activity in the ASD group compared with the typically developing group across both social and monetary reward conditions did not survive correction for multiple comparisons.

These results do not support opposing effects of social and monetary reward types, but rather point towards a general hypoactivity of ventral striatum in ASD during anticipation of rewards. This is in contrast to the hypothesis of a predominantly social motivation deficit.^1^ Instead, in line with two recent meta-analyses,^4,5^ our results suggest atypical reward processing in ASD for both social and monetary rewards. However, only one of the meta-analyses^4^ explored reward anticipation and delivery separately, and the pattern of atypicalities we observed differs from those reported there. Although the meta-analysis reports opposing patterns of ventral striatum activation for social and non-social rewards when taking into account the reward processing phase (wanting and liking of rewards), we report the same ventral striatum pattern for monetary and social rewards. We conclude that, in ASD, general hypoactivation during the anticipation of rewards reflects attenuated ‘wanting’ of rewards independent of social content. Our finding is in line with a previous study investigating negative social and monetary reinforcement^27^ and extends beyond ASD and ADHD to other conditions such as schizophrenia and bipolar disorder,^28^ pointing towards a potential shared motivational shift in these conditions that need further investigation.

Our results on reward delivery do not show substantial differences between ASD and typically developing individuals. Although this is in contrast to meta-analytic findings^4^ and previous studies showing striatal hypoactivity during monetary reward delivery^10–12^ it is in line with studies showing no significant group differences during social reward,^8,9^ monetary reward^8^ or showing differences only at an uncorrected threshold.^13^ In summary, our results suggest that both monetary and social rewards are eliciting reward-related brain activity upon delivery that is not strikingly different in individuals with ASD and typically developing individuals. Behaviourally, individuals with ASD did not differ from typically developing individuals regarding reaction times and accuracy (see Supplementary material), which is in line with previous findings, see for example references ^9,10,13,29^.

### Interpretation of our findings regarding autism

Incentive stimuli, such as the arrows used in the present study, have gained reward value (wanting) through learning processes by linking them to pleasant outcomes (liking). If individuals with ASD perceived social stimuli as less rewarding,^1^ we would expect both reduced ‘liking’ as well as reduced ‘wanting’ of social rewards in ASD, but no alterations for non-social rewards. The generally reduced activation of the reward system in the ASD group compared with the typically developing group during anticipation of rewards strongly suggest reward processing alterations in autism go beyond social stimuli. The lack of a striking difference between the typically developing and ASD groups for reward delivery indicates that alterations in wanting a reward seem to be more substantial than alterations in liking a reward. This might potentially reflect an altered link between ‘liking’ a reward and ‘wanting’ a subsequent reward, irrespective of reward type, and could suggest generally atypical reinforcement-dependent learning^30^ and/or salience processing in ASD, see for example reference.^31^ A hypothesis of generally atypical reward processing in ASD is, however, challenged by studies reporting elevated reward system responsivity in ASD to stimuli of high interest for autistic individuals or to food images.^4,9,12^ These findings indicate intact, possibly even hyperactive reinforcement-dependent learning when stimuli with high individual interest are involved. Recent models have tried to address this by proposing an imbalanced response for different reward stimuli.^2,3^ Future work is therefore needed, exploring potential changes in feedback loops underlying altered reinforcement-dependent learning in ASD using connectivity metrics and different reward types, as well as exploring links to atypical salience processing in ASD.

Although significant differences between diagnostic groups were found, we did not observe significant associations between autism trait scores (SRS-2) and functional brain activation across the whole sample or within the ASD and typically developing groups separately. In Supplementary analyses (see Supplementary Table 4) we assessed the effects of autism trait scores for MID and SID separately, but observed no significant effect in this separate analysis. Although others have found associations between dimensional autism measures and reward-related brain activity,^4,8,13,29^ our results are in line with previous studies also finding no association with dimensional autism measures.^10,12^ Previous studies argued that their null findings might be because of insufficient power and insufficient range of scores in the ASD group,^10,12^ which is less of a concern in the present findings. Recent electrophysiological findings might point towards a diverging trajectory of building reward anticipation for high versus low autistic traits, which might mask associations with autistic traits based on the time point when reward anticipation is measured.^32^

### Main findings regarding co-occurring ADHD

Elevated ADHD symptoms did not have an additive effect on reward system atypicality in ASD, in contrast to our hypothesis. Dimensional analysis of ADHD symptoms yielded no significant results. During reward anticipation, *post hoc* pairwise comparisons revealed that ventral striatum activity was reduced only in those individuals with ASD that had subthreshold levels of ADHD (ASD_–ADHD_) compared with typically developing participants, whereas those individuals with ASD that had elevated ADHD levels (ASD_+ADHD_,) did not differ significantly from the typically developing group or the ASD_–ADHD_ subgroup. During reward delivery, differences between the three groups were not strong enough to reach statistical significance when correcting for multiple comparisons. However, the direction of the effect also suggested the largest deviation for the ASD_–ADHD_ group. These results support an alternative hypothesis of ADHD symptoms partly countering ASD-related motivational atypicalities. This would be in line with a previous finding, where individuals with ASD showed generally low ventral striatum reactivity, and individuals with ADHD showed high ventral striatum reactivity to both social and monetary reward types.^13^ However, this study did not differentiate between reward anticipation and delivery.

### Interpretation of our findings regarding co-occurring ADHD

Although during monetary reward anticipation, ventral striatum hypoactivation is discussed as a fairly consistent finding in adults and adolescents with ADHD (see reference ^14^, but see also reference ^33^), findings are more inconsistent in children (for example see reference ^34^). For monetary reward delivery, increased ventral striatum activity in ADHD has been reported (see for example references ^33,35,36^ but see also reference ^29^). Importantly, information on social reward processing in ADHD is scarce. To our knowledge, the present study is the first to address co-occurring ADHD symptoms during reward anticipation and delivery separately. Instead of an additive effect of previously described atypicalities in these two conditions, our findings suggest ASD with co-occurring ADHD might represent a subgroup of individuals that, on average, shows no statistically distinct neuronal deviation from typically developing individuals nor those with ASD. This is especially important in light of the ongoing search for biomarkers underlying these neurodevelopmental conditions. It further refutes a potential concern that reward system alterations in ASD could, in truth, be driven by co-occurring ADHD. However, information on the presence of a confirmed diagnosis of ADHD was not available in our sample, and a questionnaire-derived proxy was used instead. This might have a significant impact on our findings, as ADHD-like behaviours might have been misclassified. Consequently, our finding requires further investigation using clinically confirmed information on ASD–ADHD co-occurrence.

### Limitations

Although the present study provides important insights into group-level on-average reward processing alterations in autism, a number of limitations need to be considered. First, although our findings of differences in reward processing between the ASD and typically developing groups were significant for the anticipatory phase, effect sizes were small. This likely reflects substantial between-subject heterogeneity partly attributable to the multicentre design of the study and to the intention of collecting a representative data-set reflecting the heterogeneity of ASD. We aim to further explore this heterogeneity within the LEAP sample using classification and stratification approaches in future studies.

Second, the task design did not allow for a clear separation between feedback presentation and motor response (short interstimulus interval, no jitter). Thus, we cannot rule out that findings in the delivery phase were influenced by motor activity. Third, we used static images for social reward, which likely reduced ecological validity. Fourth, task order was fixed across all participants. Thus, task order might have had an impact on the effect of stronger differential brain activation in the MID compared with the SID task. Finally, although incentive delay tasks are widely used to study reward processing, they do not allow us to pinpoint brain activity in relation to just one reward-processing aspect, but comprise multiple additional subconstructs such as reinforcement-based learning, reward valuation and reward prediction.

### Implications

In summary, the present study demonstrates a significant reduction of ventral striatum activity during the anticipation of rewards in individuals with ASD irrespective of the type of reward, and subthreshold hyperactivity of ventral striatum during the delivery of these rewards. In contrast to our hypothesis, altered reward processing was not exacerbated by elevated ADHD symptoms. This might suggest generally atypical reward processing in ASD that is partly countered by co-occurring ADHD. This provides important insights, specifically as the impact of co-occurring ADHD has not been consistently assessed in previous studies on reward processing alterations in ASD and might contribute towards the heterogeneity of findings. It might further explain discrepancies between our findings and the two meta-analyses,^4,5^ given the similar sample size while both meta-analyses did not address ADHD symptoms. Although further exploration of the underlying mechanisms is needed, the present study advances our understanding of the neural underpinnings of ASD by suggesting attenuated reward seeking independent of social content that is partly countered by co-occurring ADHD.

Future studies on reward processing, especially on reward anticipation, in ASD should thus collect and report data on ADHD symptoms and diagnosis effects. The current study presents just one step towards a broader and deeper understanding of reward processing alterations in autism, which should ultimately improve services for autistic individuals, for example by establishing alternatives to reinforcement-based interventions where needed or by including personalised rewards as alternatives to generic rewards when employing established reinforcement-based interventions.

## Data Availability

The data that support the findings of this study are currently not publicly available due to an embargo period but are available from the corresponding author upon reasonable request.

## Supplementary material

For supplementary material accompanying this paper visit https://doi.org/10.1192/bjp.2022.157. click here to view supplementary material

## References

[1] Chevallier C, Kohls G, Troiani V, Brodkin ES, Schultz RT. The social motivation theory of autism. Trends Cogn Sci 2012; 16: 231–9. 22425667 10.1016/j.tics.2012.02.007 PMC3329932

[2] Kohls G, Yerys BE, Schultz RT. Striatal development in autism: repetitive behaviors and the reward circuitry. Biol Psychiatry 2014; 76: 358–9. 25103541 10.1016/j.biopsych.2014.07.010 PMC4780436

[3] Tschida JE, Yerys BE. A systematic review of the positive valence system in autism spectrum disorder. Neuropsychol Rev 2021; 31: 58–88. 33174110 10.1007/s11065-020-09459-z

[4] Clements CC, Zoltowski AR, Yankowitz LD, Yerys BE, Schultz RT, Herrington JD. Evaluation of the social motivation hypothesis of autism: a systematic review and meta-analysis. JAMA Psychiatry 2018; 75: 797–808. 29898209 10.1001/jamapsychiatry.2018.1100 PMC6143096

[5] Janouschek H, Chase HW, Sharkey RJ, Peterson ZJ, Camilleri JA, Abel T, The functional neural architecture of dysfunctional reward processing in autism. NeuroImage Clin 2021; 31: 102700. 34161918 10.1016/j.nicl.2021.102700 PMC8239466

[6] Scott-Van Zeeland AA, Dapretto M, Ghahremani DG, Poldrack RA, Bookheimer SY. Reward processing in autism. Autism Res 2010; 3: 53–67. 20437601 10.1002/aur.122 PMC3076289

[7] Delmonte S, Balsters JH, McGrath J, Fitzgerald J, Brennan S, Fagan AJ, Social and monetary reward processing in autism spectrum disorders. Mol Autism 2012; 3: 7. 23014171 10.1186/2040-2392-3-7 PMC3499449

[8] Dichter GS, Richey JA, Rittenberg AM, Sabatino A, Bodfish JW. Reward circuitry function in autism during face anticipation and outcomes. J Autism Dev Disord 2012; 42: 147–60. 22187105 10.1007/s10803-011-1221-1 PMC8624275

[9] Kohls G, Antezana L, Mosner MG, Schultz RT, Yerys BE. Altered reward system reactivity for personalized circumscribed interests in autism. Mol Autism 2018; 9. 10.1186/s13229-018-0195-7 PMC5791309 29423135

[10] Kohls G, Schulte-Ruther M, Nehrkorn B, Muller K, Fink GR, Kamp-Becker I, Reward system dysfunction in autism spectrum disorders. Soc Cogn Affect Neurosci 2013; 8: 565–72. 22419119 10.1093/scan/nss033 PMC3682440

[11] Assaf M, Hyatt CJ, Wong CG, Johnson MR, Schultz RT, Hendler T, Mentalizing and motivation neural function during social interactions in autism spectrum disorders. NeuroImage Clin 2013; 3: 321–31. 24273716 10.1016/j.nicl.2013.09.005 PMC3815022

[12] Dichter GS, Felder JN, Green SR, Rittenberg AM, Sasson NJ, Bodfish JW. Reward circuitry function in autism spectrum disorders. Soc Cogn Affect Neurosci 2012; 7: 160–72. 21148176 10.1093/scan/nsq095 PMC3277365

[13] Kohls G, Thonessen H, Bartley GK, Grossheinrich N, Fink GR, Herpertz-Dahlmann B, Differentiating neural reward responsiveness in autism versus ADHD. Dev Cogn Neurosci 2014; 10: 104–16. 25190643 10.1016/j.dcn.2014.08.003 PMC6987952

[14] Plichta MM, Scheres A. Ventral-striatal responsiveness during reward anticipation in ADHD and its relation to trait impulsivity in the healthy population: a meta-analytic review of the fMRI literature. Neurosci Biobehav Rev 2014; 38: 125–34. 23928090 10.1016/j.neubiorev.2013.07.012 PMC3989497

[15] Grimm O, van Rooij D, Hoogman M, Klein M, Buitelaar J, Franke B, Transdiagnostic neuroimaging of reward system phenotypes in ADHD and comorbid disorders. Neurosci Biobehav Rev 2021; 128: 165–81. 34144113 10.1016/j.neubiorev.2021.06.025

[16] Chantiluke K, Christakou A, Murphy CM, Giampietro V, Daly EM, Ecker C, Disorder-specific functional abnormalities during temporal discounting in youth with attention deficit hyperactivity disorder (ADHD), autism and comorbid ADHD and autism. Psychiatry Res 2014; 223: 113–20. 24929553 10.1016/j.pscychresns.2014.04.006

[17] Loth E, Charman T, Mason L, Tillmann J, Jones EJH, Wooldridge C, The EU-AIMS longitudinal European autism project (LEAP): design and methodologies to identify and validate stratification biomarkers for autism spectrum disorders. Mol Autism 2017; 8: 24. 28649312 10.1186/s13229-017-0146-8 PMC5481887

[18] Plichta MM, Schwarz AJ, Grimm O, Morgen K, Mier D, Haddad L, Test-retest reliability of evoked BOLD signals from a cognitive-emotive fMRI test battery. NeuroImage 2012; 60: 1746–58. 22330316 10.1016/j.neuroimage.2012.01.129

[19] Charman T, Loth E, Tillmann J, Crawley D, Wooldridge C, Goyard D, The EU-AIMS longitudinal European autism project (LEAP): clinical characterisation. Mol Autism 2017; 8: 27. 28649313 10.1186/s13229-017-0145-9 PMC5481972

[20] Lord C, Rutter M, Le Couteur A. Autism diagnostic interview-revised: a revised version of a diagnostic interview for caregivers of individuals with possible pervasive developmental disorders. J Autism Dev Disord 1994; 24: 659–85. 7814313 10.1007/BF02172145

[21] Lord C, Risi S, Lambrecht L, Cook EH Jr., Leventhal BL, DiLavore PC, The autism diagnostic observation schedule-generic: a standard measure of social and communication deficits associated with the spectrum of autism. J Autism Dev Disord 2000; 30: 205–23. 11055457

[22] Constantino JN, Davis SA, Todd RD, Schindler MK, Gross MM, Brophy SL, Validation of a brief quantitative measure of autistic traits: comparison of the social responsiveness scale with the autism diagnostic interview-revised. J Autism Dev Disord 2003; 33: 427–33. 12959421 10.1023/a:1025014929212

[23] DuPaul GJ, Power TJ, Anastopoulos AD, Reid R. ADHD Rating Scale—IV: Checklists, Norms, and Clinical Interpretation. Guilford Press, 1998.

[24] Moessnang C, Schafer A, Bilek E, Roux P, Otto K, Baumeister S, Specificity, reliability and sensitivity of social brain responses during spontaneous mentalizing. Soc Cogn Affect Neurosci 2016; 11: 1687–97. 27445211 10.1093/scan/nsw098 PMC5091688

[25] Plichta MM, Grimm O, Morgen K, Mier D, Sauer C, Haddad L, Amygdala habituation: a reliable fMRI phenotype. NeuroImage 2014; 103: 383–90. 25284303 10.1016/j.neuroimage.2014.09.059

[26] Faul F, Erdfelder E, Lang AG, Buchner A. G*Power 3: a flexible statistical power analysis program for the social, behavioral, and biomedical sciences. Behav Res Methods 2007; 39: 175–91. 17695343 10.3758/bf03193146

[27] Damiano CR, Cockrell DC, Dunlap K, Hanna EK, Miller S, Bizzell J, Neural mechanisms of negative reinforcement in children and adolescents with autism spectrum disorders. J Neurodev Disord 2015; 7: 12. 25829969 10.1186/s11689-015-9107-8 PMC4379694

[28] Schwarz K, Moessnang C, Schweiger JI, Baumeister S, Plichta MM, Brandeis D, Transdiagnostic prediction of affective, cognitive, and social function through brain reward anticipation in schizophrenia, bipolar disorder, major depression, and autism spectrum diagnoses. Schizophr Bull 2020; 46: 592–602. 31586408 10.1093/schbul/sbz075 PMC7147576

[29] van Dongen EV, von Rhein D, O'Dwyer L, Franke B, Hartman CA, Heslenfeld DJ, Distinct effects of ASD and ADHD symptoms on reward anticipation in participants with ADHD, their unaffected siblings and healthy controls: a cross-sectional study. Mol Autism 2015; 6: 48. 26322219 10.1186/s13229-015-0043-y PMC4551566

[30] Wise RA. Dopamine, learning and motivation. Nat Rev Neurosci 2004; 5: 483–94. 15152198 10.1038/nrn1406

[31] Uddin LQ, Supekar K, Lynch CJ, Khouzam A, Phillips J, Feinstein C, Salience network-based classification and prediction of symptom severity in children with autism. JAMA Psychiatry 2013; 70: 869–79. 23803651 10.1001/jamapsychiatry.2013.104 PMC3951904

[32] Matyjek M, Bayer M, Dziobek I. Autistic traits affect reward anticipation but not reception. Sci Rep 2020; 10: 8396. 32440002 10.1038/s41598-020-65345-x PMC7242422

[33] von Rhein D, Cools R, Zwiers MP, van der Schaaf M, Franke B, Luman M, Increased neural responses to reward in adolescents and young adults with attention-deficit/hyperactivity disorder and their unaffected siblings. J Am Acad Child Adolesc Psychiatry 2015; 54: 394–402. 25901776 10.1016/j.jaac.2015.02.012 PMC4417499

[34] van Hulst BM, de Zeeuw P, Bos DJ, Rijks Y, Neggers SF, Durston S. Children with ADHD symptoms show decreased activity in ventral striatum during the anticipation of reward, irrespective of ADHD diagnosis. J Child Psychol Psychiatry 2017; 58: 206–14. 27678006 10.1111/jcpp.12643

[35] Paloyelis Y, Mehta MA, Faraone SV, Asherson P, Kuntsi J. Striatal sensitivity during reward processing in attention-deficit/hyperactivity disorder. J Am Acad Child Adolesc Psychiatry 2012; 51: 722–32.e9. 22721595 10.1016/j.jaac.2012.05.006 PMC3763946

[36] Furukawa E, Bado P, Tripp G, Mattos P, Wickens JR, Bramati IE, Abnormal striatal BOLD responses to reward anticipation and reward delivery in ADHD. PLoS One 2014; 9: e89129. 24586543 10.1371/journal.pone.0089129 PMC3935853

